# The Anterior Chamber Injection of Moxifloxacin Injection to Prevent Endophthalmitis after Cataract Surgery: A Meta-analysis

**DOI:** 10.1155/2020/7242969

**Published:** 2020-08-25

**Authors:** Xiao-lei Wang, Xiao-yong Huang, Zhen Wang, Wei Sun

**Affiliations:** ^1^Department of Ophthalmology, Southwest Hospital, Army Medical University, Chongqing 400038, China; ^2^Department of Emergency, Southwest Hospital, Army Medical University, Chongqing 400038, China

## Abstract

**Purpose:**

A meta-analysis was performed to compare the efficacy of an anterior chamber injection of moxifloxacin in the prevention of endophthalmitis after cataract surgery.

**Methods:**

A computer-based search of PubMed, Embase, the Cochrane Library, and the Clinical Trial database for articles related to anterior intraventricular injection of moxifloxacin for the prevention of endophthalmitis after cataract surgery was performed through April 2019. Study selection, data exclusion, and quality assessment were performed by two independent observers. Statistical analysis for the meta-analysis was performed by RevMan5.3 software.

**Results:**

Eight studies were included, with a total of 123,819 eyes. The meta-analysis showed that an anterior chamber injection of moxifloxacin can prevent the incidence of endophthalmitis after cataract surgery (OR = 0.29, 95% CI (0.15, 0.56), *P*=0.0002), and the difference was statistically significant. There were no significant differences between the moxifloxacin injection and nonmoxifloxacin injection groups in regard to UCVA (log MAR) (SMD = −0.13, 95% CI (−0.62, 0.35), *P*=0.60), BCVA (log MAR) (SMD = −0.27, 95% CI (−1.28, 0.74), *P*=0.60), IOP (SMD = −0.04, 95% CI (−0.02, 0.01), *P*=0.22), corneal edema (OR = 1.03, 95% CI (0.23, 4.69), *P*=0.97), CCT (SMD = −0.01, 95% CI (−0.07, 0.05), *P*=0.77), or ECD (SMD = 0.00, 95% CI (−0.06, 0.07), *P*=0.94).

**Conclusion:**

An anterior chamber injection of moxifloxacin can effectively prevent the incidence of endophthalmitis after cataract surgery, while the moxifloxacin injection and nonmoxifloxacin injection groups had similar results in regard to UCVA (log MAR), BCVA (log MAR), IOP, corneal edema, CCT, and ECD.

## 1. Background

Endophthalmitis is one of the most serious complications after cataract surgery. Although rare, once it occurs, it is a complication that leads to poor visual prognosis [1]. According to the related literature [2], the incidence of endophthalmitis after cataract surgery is 0∼0.63%. There are several ways to prevent endophthalmitis, such as the use of povidone iodine, which can reduce the incidence of endophthalmitis [3]; antibiotic eye drops; subconjunctivals; anterior chamber injection; and antibacterial drug flushing [4–6]. In 2007, the European Association of Cataract and Refractive Surgeons (ESCRS) published a multicenter clinical trial that demonstrated the benefits of an anterior chamber injection of cefuroxime in preventing postoperative endophthalmitis [7]. In the recent years, the injection of antibacterial drugs in the anterior chamber has received increasing attention, and some related retrospective clinical studies and systematic reviews have been published. [8–10] However, with the increase in bacterial resistance, antibacterial drugs such as vancomycin and moxifloxacin have gradually begun to be used clinically to prevent postoperative endophthalmitis [9, 11]. However, cefuroxime is less sensitive to drug-resistant bacteria, and the use of vancomycin may cause hemorrhagic occlusive retinal vasculitis. Therefore, it is more appropriate to prevent postoperative endophthalmitis via injection with moxifloxacin. However, due to the influence of the baseline characteristics, follow-up time, and research institutions of the included populations, the conclusions among studies have not been uniform. For this reason, we conducted this meta-analysis to provide a reference for the rational use of antibiotics in the perioperative period.

## 2. Methods

This review was conducted in accordance with the Preferred Reporting Items for Systematic Reviews and Meta-Analyses (PRISMA) guidelines and the recommendations of the Cochrane Collaboration.

### 2.1. Search Strategy

A computer search using PubMed, Embase, the Cochrane Library, and the Clinical Trial database for the intra-anterior injection of moxifloxacin to prevent endophthalmitis after cataract surgery was performed; the search time limit was from the establishment of the database to April 2019. The search terms were moxifloxacin, moxifloxacin hydrochloride, ophthalmologic surgical procedures, cataract extraction, vitrectomies, keratoplasties, intraocular lens implantations, glaucoma procedures, strabotomies, retinal detachment repair, laser in situ keratomileusis, and laser-assisted subepithelial keratectomy.

### 2.2. Eligibility Criteria

(1) Study designs: RCTs, case-control studies, or cohort studies. (2) Types of participants: eligible for cataract surgery; no limitation regarding age, sex, and race; and absence of moxifloxacin allergy, traumatic cataract with perforation of the eye, cataract surgery combined with other operations (such as glaucoma filtration surgery, vitreoretinal surgery, or corneal surgery), eye or periocular infection, advanced glaucoma, and severe ocular surface disease. (3) Intervention: anterior chamber injection of moxifloxacin, frequency of administration, dose, and concentration. (4) Outcomes: the incidence of endophthalmitis, uncorrected visual acuity (UCVA), best corrected visual acuity (BCVA), intraocular pressure (IOP), corneal edema, corneal center thickness (CCT), and corneal endothelial cell density (ECD).

### 2.3. Exclusion Criteria

The exclusion criteria were as follows: (1) animal experiments; (2) repeated publications; (3) the literature for which data could not be extracted; and (4) abstracts, reviews, or conference literature.

### 2.4. Data Extraction

Two researchers used a three-step method to independently screen the literature, in case of disagreement. First, the topic and abstract were read, the irrelevant literature was excluded, and then, the full text of the remaining articles was read to determine whether they were ultimately included. If there were differences, they were discussed with a third party. The data extraction included the following: the first author and study time, age, sex, follow-up time, treatment plan, timing of administration, number of eyes included in the study, research type, and outcome indicators.

Regarding quality assessment of RCTs, the Cochrane risk of bias tool was used to perform a methodological quality assessment of RCTs. Assessment items include randomization, blinding, allocation concealment, data integrity, selective reporting bias, and other sources of bias. Each item was evaluated as “high,” “low,” or “unclear.” For non-RCTs, the literature quality was evaluated for case-control studies and cohort studies using the Newcastle–Ottawa Scale (NOS) for literature quality assessment.

### 2.5. Data Synthesis and Statistical Analysis

Statistical analysis was performed using RevMan5.3 software. The continuous variables used the standardized mean difference (SMD) and its 95% confidence interval (CI) as the statistical analysis value; the odds ratio (OR) and its 95% CI were selected for the two categorical variables. The heterogeneity between studies was investigated by the *Q* test and *I*^2^ test. If *P* was ≤0.1 or *I*^2^ was ≥50%, heterogeneity was considered significant. Sensitivity analysis was performed to determine whether the heterogeneity decreased after each study was excluded. If the heterogeneity was not reduced, a subgroup analysis was performed based on the clinical characteristics of these studies. In the sensitivity analysis and subgroup analysis, if the heterogeneity was not reduced, the random effects model was used and analyzed by the Mantel–Haenszel method. If there was no heterogeneity between studies, indicated by *P* > 0.1 or *I*^2^ < 50%, the analysis was performed using a fixed effect model. All the combined results were statistically significant according to *P* < 0.5. A publication bias test was performed using a funnel plot.

## 3. Result

### 3.1. Search Results

**Figure 1:**
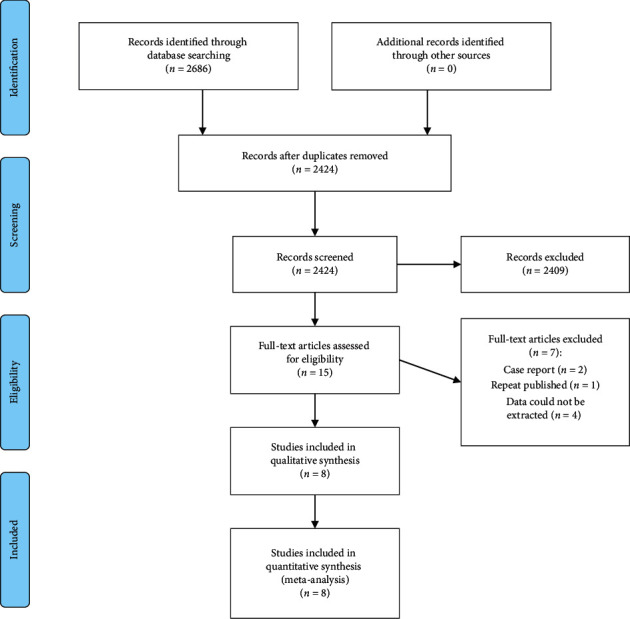
Flowchart of selection of studies for inclusion in the meta-analysis.

A total of 2,686 articles were collected through relevant database searches and other resources, and 262 duplicates were deleted. After exclusion according to the topic, abstract, and intensive reading of the full text, 8 articles were finally included [11–18]. The literature was analyzed by Meta. The literature flow chart is shown in Figure 1.

### 3.2. Characteristics of Included Studies

This meta-analysis included 8 studies [11–18], with 123,819 eyes; 2 studies were RCTs [11, 17], 2 studies were case-control studies [12, 13], and 4 studies were cohort studies [14–16, 18]. Two studies [11, 14] were conducted in Brazil, 1 study [17] was conducted in the USA, 1 study [12]was conducted in Turkey, 1 study [13] was conducted in Canada, 1 study [18] was conducted in Colombia, and 2 studies [15, 16] were conducted in Japan. The follow-up time ranged from 2 weeks to 1 year. The basic characteristics of the literature are shown in Table 1.

#### 3.2.1. Methodological Quality Evaluation

**Figure 2:**
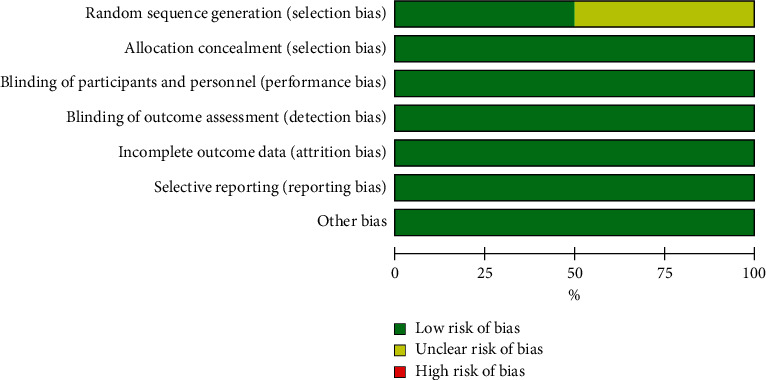
Risk of bias graph.

**Figure 3:**
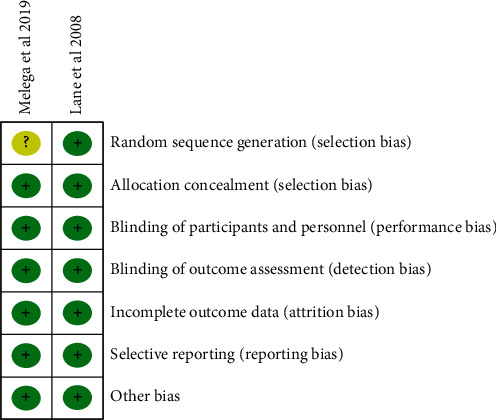
Risk of bias summary.

Two studies [11, 17] were RCTs, and we used the Cochrane risk of bias tool to assess these studies; the RCT quality evaluation results are shown in Figures 2 and 3. For non-RCTs [12–16, 18], we used the Newcastle–Ottawa scoring system to evaluate the quality of the literature. The total scores were 1 to 3, 4 to 6, and 7 to 9, representing low-, medium-, and high-quality studies, respectively. Two articles [14, 18] were of high quality, and 4 [12, 13, 15, 16] articles were of medium quality. The results of the non-RCT literature quality evaluation are shown in Table 2.

### 3.3. The Incidence of Endophthalmitis

**Figure 4:**
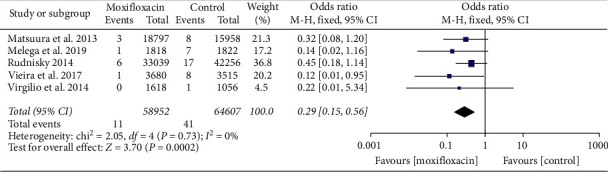
Forest plot of the incidence of endophthalmitis.

We included 5 articles in this analysis [11, 13–15, 18]. There was no heterogeneity between the studies (*I*^2^ = 0%, *P*=0.73), and a fixed effect model was used. Meta-analysis showed that anterior chamber injection of moxifloxacin could prevent the incidence of endophthalmitis after cataract surgery (OR = 0.29, 95% CI (0.15, 0.56), *P*=0.0002), and the difference was statistically significant, as shown in Figure 4.

### 3.4. UCVA

**Figure 5:**
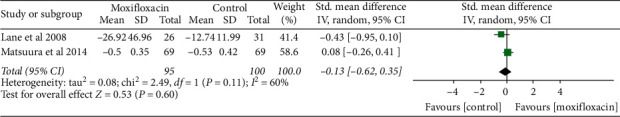
Forest plot of UCVA.

Two articles [15, 17] were included that reported UCVA (log MAR). The meta-analysis showed no significant difference between the moxifloxacin injection and nonmoxafloxacin injection (SMD = −0.13, 95% CI (−0.62, 0.35), *P*=0.60). Since *I*^2^ = 60%, the random effects model was used for analysis, as shown in Figure 5.

### 3.5. BCVA

**Figure 6:**
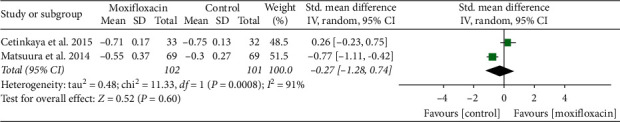
Forest plot of BCVA.

Two studies [12, 15] reported BCVA (log MAR). The meta-analysis showed no significant difference between the moxifloxacin injection and nonmoxafloxacin injection (SMD = −0.27, 95% CI (−1.28, 0.74), *P*=0.60). Since *I*^2^ = 91%, the random effects model was used for analysis, as shown in Figure 6.

### 3.6. IOP

**Figure 7:**
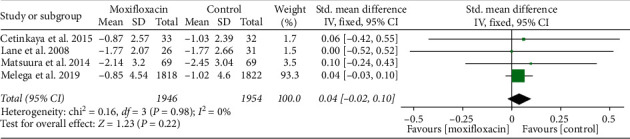
Forest plot of IOP.

Four studies [11, 12, 15, 17] reported IOP with no heterogeneity between studies (*I*^2^ = 0%, *P*=0.98) using a fixed effect model. The meta-analysis showed no significant difference between the moxifloxacin injection and nonmoxafloxacin injection (SMD = −0.04, 95% CI (−0.02, 0.01), *P*=0.22); see Figure 7.

### 3.7. Corneal Edema

**Figure 8:**
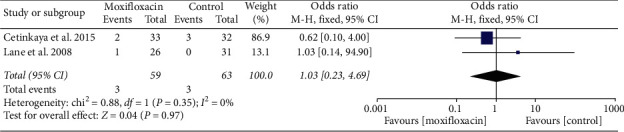
Forest plot of corneal edema.

Two studies [12, 17] reported corneal edema, with no heterogeneity between studies (*I*^2^ = 0%, *P*=0.35); therefore, a fixed-effects model was used. The meta-analysis showed no significant difference between the moxifloxacin injection and nonmoxafloxacin injection (OR = 1.03, 95% CI (0.23, 4.69), *P*=0.97, Figure 8).

### 3.8. CCT

**Figure 9:**
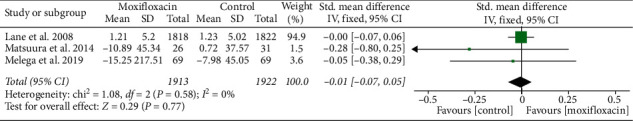
Forest plot of CCT.

Three studies [11, 15, 17] reported CCT, and there was no heterogeneity between the studies (*I*^2^ = 0%, *P*=0.58); therefore, a fixed effect model was used. The meta-analysis showed no significant difference between the moxifloxacin injection and nonmoxifloxacin injection (SMD = −0.01, 95% CI (−0.07, 0.05), *P*=0.77, Figure 9).

### 3.9. ECD

**Figure 10:**
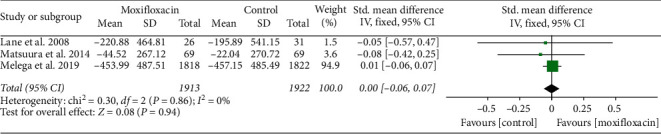
Forest plot of ECD.

Three studies [11, 15, 17] reported ECD, and there was no heterogeneity between the studies (*I*^2^ = 0%, *P*=0.86); therefore, a fixed effect model was used. The meta-analysis showed no significant difference between the moxifloxacin injection and nonmoxifloxacin injection (SMD = 0.00, 95% CI (−0.06, 0.07), *P*=0.94, Figure 10).

## 4. Discussion

The incidence of endophthalmitis after cataract surgery is 0.012∼0.053% in developed countries. [19] The incidence in large ophthalmology institutions in China is approximately 0.033% [20], and the incidence in small ophthalmology institutions has increased to 0.11% [21]. Because the incidence of endophthalmitis is extremely low, it is difficult to verify which preventive measures are the most effective through large RCTs, so most of the current research is observational in nature. Once endophthalmitis occurs, it can be life threatening. Perioperative use of antibiotic eye drops and preoperative conjunctival vesicle povidone iodine disinfection have been indicated to be the most effective measures to prevent endophthalmitis after cataract surgery. These measures have been considered the medical standards, and the anterior chamber injection of antibiotics' safety and efficacy have also been popular research topics. However, the most reasonable use of antibiotics as a preventive measure during the perioperative period is still controversial. Different methods of administration, timing, and the course of treatment of anti-inflammatory drugs for postoperative endophthalmitis have been proposed by ophthalmologists, but there is no consensus. After the European ESCRS study, the anterior chamber injection of cefuroxime has been widely recognized, and with increasing clinical bacterial resistance, some medical institutions have begun to use effective broad-spectrum antibiotics, such as moxifloxacin and vancomycin. The anterior chamber injection of strong antibiotics has been used to prevent endophthalmitis after cataract surgery. In clinical applications, the preparation of cefuroxime in the pharmacy will increase the risk of infection and toxic anterior segment syndrome. The temporary preparation in the operating room is prone to dose error, while the direct injection of commercial cefuroxime is only available in Europe [22]. In addition, in a small number of cases, there is an allergic reaction associated with cefuroxime or transient macular edema caused by overdose [23, 24]. The use of vancomycin may cause hemorrhagic occlusive retinal vasculitis.

The results of this meta-analysis showed that the anterior chamber injection of moxifloxacin can prevent the incidence of endophthalmitis after cataract surgery (OR = 0.29, 95% CI (0.15, 0.56), *P*=0.0002), and the difference was statistically significant. Regarding other indicators (UCVA (log MAR), BCVA (log MAR), IOP, corneal edema, CCT, and ECD), there was no significant difference between the moxifloxacin injection group and the nonmoxifloxacin injection group. Moxifloxacin has a wide range of antibacterial activities and is effective against *Pseudomonas aeruginosa*. Moxifloxacin is safe for patients allergic to penicillin and cephalosporin. It can be directly injected into the anterior chamber without preservative-containing eye drops. The operation is the simplest. Combined with the results of this study, it can be considered clinically safe to use the anterior chamber injection of moxifloxacin to prevent endophthalmitis after cataract surgery. However, due to the current small number of clinical studies and the lack of prospective RCTs, this study included only two RCTs; the rest were non-RCT studies, and there was a lack of direct comparison between moxifloxacin and cefuroxime. Therefore, large-sample, multicenter, high-quality RCTs are needed in the future to provide higher quality evidence.

The study was limited by the following factors: (1) Because the incidence of endophthalmitis after cataract surgery is extremely low, most studies are observational; however, there is much evidence that the anterior chamber injection of moxifloxacin can prevent endophthalmitis. The incidence of posterior endophthalmitis, which is not used by clinicians, was not possible to assess mainly due to a lack of high-quality evidence from RCTs. (2) The follow-up time of some of the included studies was short, so we may have underestimated drug-induced adverse events. (3) Heterogeneity is unavoidable due to factors such as different drug administration schedules, different follow-up times, and differences in the population. (4) In addition to the significant difference in the incidence of endophthalmitis in this study, the difference in other indicators was not statistically significant, which may be due to the lack of included studies and the small sample size. Therefore, the advantages of the moxifloxacin injection in these areas have not been shown.

## 5. Conclusions

The results of this meta-analysis showed that compared with a nonmoxifloxacin injection, an anterior chamber injection of moxifloxacin was effective in preventing the incidence of endophthalmitis after cataract surgery, and the moxifloxacin injection exhibited in similar results as a nonmoxifloxacin injection in UCVA (log MAR), BCVA (log MAR), IOP, corneal edema, CCT, and ECD. Therefore, to obtain more meaningful results, a larger sample size RCT should be performed.

## Figures and Tables

**Table 1:** Characteristics of the included studies (*n* = 8).

Study	Site	Designs	The number of the eyes		Age		Treatment options	Follow-up	Outcomes
			Moxifloxacin	Control	Moxifloxacin	Control			
Melega et al. [11]	Brazil	RCT	1818	1822	68.50 ± 9.72	68.49 ± 9.63	0.5% moxifloxacin	6 w	ECD, IOP, CCT, and the incidence of endophthalmitis
Lane et al. [17]	USA	RCT	26	31	74 ± 9.3	74 ± 9.3	0.5% moxifloxacin	3 m	UCVA, IOP, ECD, and CCT
Cetinkaya et al. [12]	Turkey	Case-control study	33	32	64.81 ± 11.61	65.43 ± 11.10	0.5% moxifloxacin	1 y	BCVA, IOP, and corneal edema
Rudnisky et al. [13]	Canada	Case-control study	33039	42256	NA	NA	0.5% moxifloxacin	6 w	The incidence of endophthalmitis
Virgilio et al. [18]	Colombia	Cohort study	1618	1056	67.2 ± 11.3	67.2 ± 11.3	0.5% moxifloxacin	2 w	The incidence of endophthalmitis
Matsuura et al. [15]	Japan	Cohort study	69	69	71.9 ± 7.5	71.9 ± 7.5	0.5% moxifloxacin	3 m	UCVA, BCVA, IOP, ECD, and CCT
Matsuura et al., [16]	Japan	Cohort study	18797	15958	NA	NA	0.1%, 0.3%, and 0.5% moxifloxacin	1 m	The incidence of endophthalmitis
Vieira et al. [14]	Brazil	Cohort study	3680	3515	67.7 ± 9.03	68.1 ± 8.92	0.5% moxifloxacin	1 m	The incidence of endophthalmitis

Abbreviations: BCVA, best corrected visual acuity; UCVA, uncorrected visual acuity; IOP, intraocular pressure; ECD, corneal endothelial cell density; CCT, central corneal thickness; NA, data not available; y, year; m, month; w, week.

**Table 2:** Quality assessment of included observational studies based on the Newcastle–Ottawa scale.

Study	Crowd selectivity (4 points)	Comparability (2 points)	Exposure evaluation (3 points)	Total (9 points)
Cetinkaya et al. [12]	3	0	3	6
Rudnisky [13]	3	0	3	6
Galvis et al. [18]	3	1	3	7
Matsuura et al. [15]	3	0	3	6
Matsuura et al. [16]	3	0	3	6
Virgilio et al. [18]	3	1	3	7

## Data Availability

The data used to support this study were from NCBI PubMed. The data sets used and/or analyzed during the present study are available from the corresponding author on reasonable request.
